# Environmental Factors Influencing Stress Corrosion Cracking Behavior of Austenitic Stainless Steels in Simulated Seawater

**DOI:** 10.3390/ma18184317

**Published:** 2025-09-15

**Authors:** Chun-Ping Yeh, Kun-Chao Tsai, Jiunn-Yuan Huang

**Affiliations:** National Atomic Research Institute (NARI), 1000 Wenhua Rd., Longtan District, Taoyuan City 32546, Taiwan

**Keywords:** marine environment, chloride concentration, stainless steel, SCC, dust

## Abstract

Grade 304L stainless steel canisters are susceptible to crevice corrosion in marine environments. In the present study, white emery was utilized to create a simulation of dust accumulation. The corrosion testing was conducted at two distinct temperatures (35 °C and 45 °C) and three levels of relative humidity (45%, 55%, and 70% relative humidity). The chloride deposition density levels tested were 0.1 g/m^2^ and 1 g/m^2^. The test durations were 8000 h and 23,000 h. It is evident that with a chloride deposition density of 0.1 g/m^2^ at a temperature of 45 °C and a relative humidity of 70%, the onset of stress corrosion cracking (SCC) occurred after 8000 h in the white emery deposition tests. In contrast, at a 1 g/m^2^ chloride deposition density, the polytetrafluoroethylene (PTFE) crevice former test specimen exhibited continuous transgranular SCC within the same period. These quantitative findings emphasize the critical roles of salt load and environmental severity in the initiation of SCC.

## 1. Introduction

It is critical to ensure the durability of dry storage canisters to ensure the safe and secure management of spent nuclear fuel (SNF), particularly in facilities located near coastal regions. Austenitic stainless steels (ASS), particularly the 304L and 316L grades, are frequently utilized in the fabrication of these canisters due to their proven superiority in terms of resistance to corrosion [1]. However, these materials are prone to chloride-induced SCC (CISCC), a significant degradation mechanism that is further exacerbated by salt contamination from airborne particles in marine environments [2,3]. The process commences with the dissipation of heat from the decaying SNF, which reduces the surface temperature of the canisters to below 100 °C. This initiates the deliquescence of the salts that have been deposited, resulting in the production of a chloride-rich aqueous layer that promotes localized corrosion [4,5]. In order to guarantee the durability and safety of these storage systems, it is vital to gain a comprehensive understanding of the contributing environmental factors that drive CISCC.

Environmental factors, including chloride concentration, relative humidity (RH), and temperature, perform a pivotal function in the progression of CISCC. It has been demonstrated that elevated temperatures enhance corrosion kinetics and diffusion, while a high RH sustains a thin electrolyte layer, intensifying anodic dissolution and crack propagation. The Nuclear Regulatory Commission (NRC) has identified CISCC as a pivotal challenge threatening the safe long-term operation of dry storage systems for SNF [6].

Numerous researchers have studied the corrosive deterioration of stainless steel in marine surroundings. Kim et al. investigated the CISCC susceptibility of 0.6–1 M NaCl_2_ deposited on 304L–ER308L welded plates at 50–60 °C with 20–30% RH. The results demonstrated that lowering the temperature from 60 °C to 50 °C did not prevent CISCC. Conversely, a reduction in humidity to 20% RH effectively inhibited the formation of CISCC. This suggests that the inhibition of NaCl deliquescence under conditions of reduced humidity was insufficient to promote CISCC [3]. Zhao et al. conducted an analysis of the corrosion behavior of 2205 duplex SS under conditions involving the U-bending deformation and the application of MgCl_2_ droplets subjected to wet–dry cycling [7]. Dhaiveegan et al. conducted a three-year investigation into the corrosion behavior of 304 and 316L stainless steels when exposed to the Indian marine environment in the Chennai region. Based on their report, 304 SS does not demonstrate behaviors associated with passivation, whereas 316L SS reveals such behaviors in the marine surroundings of Chennai, characterized by high relative humidity [8]. Burkert et al. reported that stainless steel specimens with crevices exhibited considerably deeper pits when exposed to a marine environment [9]. Lv et al. reported that the initiation of pits is related to the dissolution of MnS inclusions [10]. In a simulated marine environment, the maximum pit depth of 304 stainless steel increased with corrosion time [10].

Austenitic stainless steel canister materials are susceptible to CISCC when exposed to chloride-containing sea salts due to the formation of a film of deliquescent salts on the canister surface that can retain moisture [11,12]. Deliquescent sea salts contain chloride ions that are highly aggressive. They induce the degradation of the passive film and the initiation of SCC. A hypothesis has been proposed that links the crevice corrosion characteristics of stainless steel to the process of passive film development within crevices [13]. As the chloride concentration increases, the stability of the passive film decreases, leading to more severe crevice corrosion phenomena [14]. It has been demonstrated that the canisters undergo a gradual accumulation of dust on their surfaces with the presence of chloride salts over time. The deposition of dust on the canister surface with the presence of chloride salts establishes an ambient environment favorable for water accumulation and subsequent localized corrosion, such as crevice corrosion.

Crevice corrosion has been shown to enhance CISCC by means of trapping deposits of chloride on the surface of the canister. Crevice corrosion initiation is affected by aggressive Cl^−^ ions, the characteristics of the crevice, and the ambient temperature. The characteristics of the crevice are established in that the canister is in physical contact with the storage module’s structural support, a feature present in both vertical and horizontal canister configurations [15]. The behavior of crevice corrosion can be explained by means of the mechanism of critical crevice solution (CCS). The depletion of oxygen in the crevice is believed to be the underlying cause of the acidification of the solution, the subsequent breakdown of the passive film, and the initiation of crevice corrosion behavior. It has been demonstrated that this mechanism is the result of the effect of aggressive ions, such as chloride ions. Accumulation of these ions in the crevice is followed by depassivation, resulting from the active dissolution of the base metal [16,17]. The susceptibility of austenitic stainless steels to SCC has been well documented, particularly in aggressive surroundings, for example, Cl^−^ ions, which can result in CISCC [18,19,20]. The increased volumetric presence of corrosion products at crack sites could create localized stresses that enhance the initiation of stress corrosion cracking. Mayuzumi et al. revealed that stress corrosion cracking initiation occurs at the bottom of crevice corrosion areas in the presence of chloride ions [21]. Tani and colleagues suggested that crevice corrosion leads to SCC initiation on surfaces subjected to saline particles [22].

The chloride deposition density on canister surfaces can be estimated by analyzing the sea salt deposit’s areal density and the local environmental conditions. Guo et al. reported on the corrosion behavior of 304 stainless steel in the presence of atmospheric conditions involving a mixture of sodium chloride (NaCl) and magnesium chloride (MgCl_2_) at a temperature of 21 °C and a relative humidity of 45%. It was determined that pits of a bowl-shaped configuration form in solutions that contain a mixture of salts, while crevice corrosion occurs beneath NaCl crystals [23]. Dong et al. examined the stress corrosion cracking susceptibility of MgCl_2_, depositing it on 316L SS at 75 °C and 70% RH. It was determined that transgranular SCC (TGSCC) occurred at pit edges in the welded region [24]. The influence of salt on CISCC in SS304L at 90 °C and 70% RH was examined by Scatigno et al. At a 1.7 g/m^2^ chloride deposition density of MgCl_2_, CISCC was found in the environment [25]. Shoji and colleagues investigated the effect of various environmental factors on the initiating behavior of CISCC. Specifically, they examined how relative humidity, temperature, and chloride deposition density influenced the initiation process. They concluded that the presence of MgCl_2_, a constituent of sea salt, has been demonstrated to promote the occurrence of low-temperature CISCC in SS304L and SS316L [26], and their works were summarized by Scatigno et al. [27]. Cook et al. provided a comprehensive summary of the ranges of relative humidity where CISCC occurred, along with the chloride deposition density of MgCl_2_ and seawater at 25 g/m^2^, based on the findings of Shoji [28]. Prosek et al. examined the CISCC phenomenon in SS304 and SS316L at a 260 g/m^2^ chloride concentration, which was achieved by the deposition of MgCl_2_ droplets [29]. In addition, based on the work of Ornek et al., the chloride deposition density at the crack tips of SS304L was found to be saturated during salt loading tests. Accordingly, the level of surface chloride did not have a significant impact on the rate of crack propagation [30].

The Nuclear Regulatory Commission (NRC) conducted experiments using 304 stainless steel U-bend specimens at temperatures ranging from 27 °C to 60 °C. According to the findings of the NRC, the chloride deposition density threshold for initiating stress corrosion cracking was determined to be 0.1 g/m^2^ [31]. Furthermore, the chloride deposition density measured on surfaces of the canister exposed to sea salt was found to be merely 1 g/m^2^, which is considerably lower than the expected value over a 19-year period [32]. The Central Research Institute of Electric Power Industry (CRIEPI) subjected 304L stainless steel samples to a 50 °C and 35% RH testing environment for over 2000 h. CRIEPI revealed that the chloride deposition density threshold for SCC initiation was 0.8 g/m^2^ [33]. Furthermore, the canister surface temperature was 40–52 °C [26], and the inlet temperature of the canister was 35 °C where surveillance coupons were located [34].

Qiao et al. demonstrated the SCC phenomenon in 321 SS when exposed to a MgCl_2_ solution. The interaction between primary cracks and discontinuous microcracks has been found to increase the effective stress intensity factor. Consequently, this interaction promotes crack coalescence, thereby resulting in the mechanical rupture of the ligaments that exist between the aforementioned cracks in stainless steel [35]. Masuda et al. examined the stress corrosion cracking behavior of 304 stainless steel, focusing on its slip deformation, pit growth, and surface potential distribution. SCC testing was executed at temperatures of 70 °C and relative humidity levels of 28% utilizing MgCl_2_ droplets. Usually, discrete cracks were detected in the region proximate to the crack tip [36].

The accumulation of dust can lead to a notable increase in surface roughness. This phenomenon is associated with the formation of more profound grooves on the surface of the material, where a high accumulation of dust and ion concentrations is observed. Consequently, this can result in the initiation of corrosion at relatively early stages [37]. In addition, an elevated roughness promotes the interaction between the corrosive agents present in moisture and the metal surface.

Despite the extensive research conducted on CISCC under atmospheric chloride de posits, there is a lack of studies employing a synthetic, high-purity particulate analogous to dust-particle effects. The existing literature is deficient in its lack of a reproducible surrogate with well-controlled size, shape, and mass. These elements are critical for the systematic evaluation of particulate-driven crevice corrosion phenomena. In this study, white emery was utilized as a model dust layer. Therefore, this study proposes a novel approach that involves the integration of a controlled white emery surrogate with well-defined environmental factors. The objective of this study is to address the critical knowledge gap concerning dust–salt particulate-driven CISCC in nuclear-grade austenitic stainless steel canisters.

## 2. Experimental Procedure

**Figure 1:**
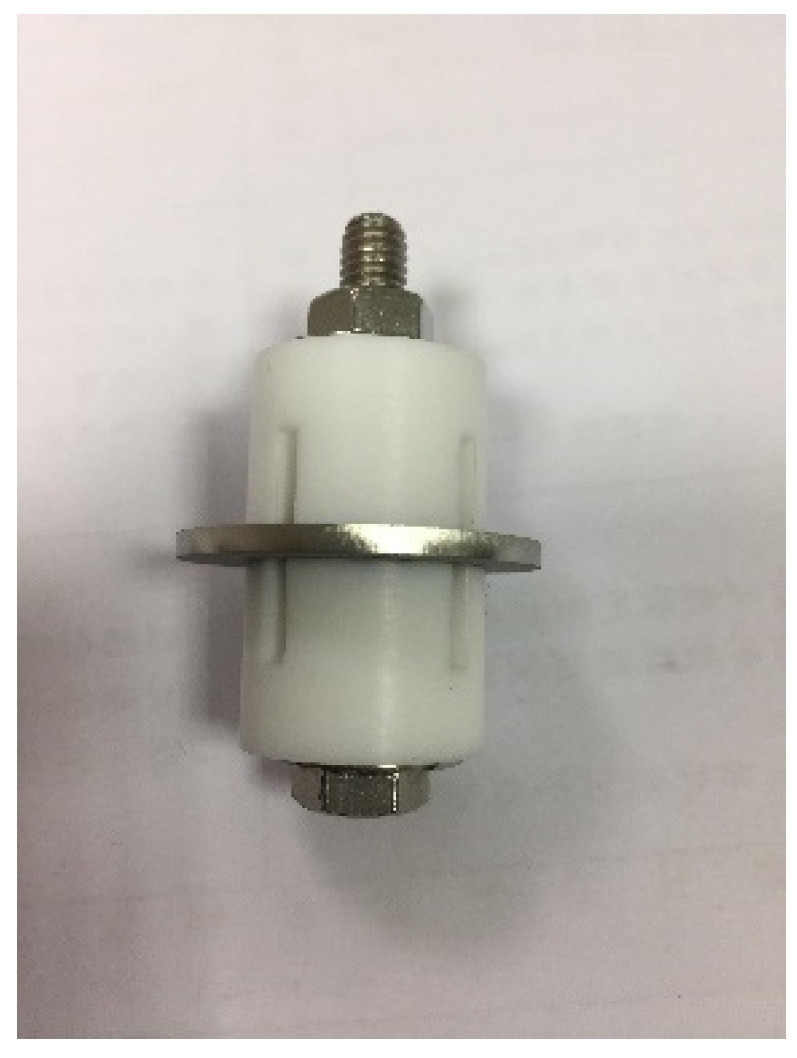
Assembled image of the 304L stainless steel specimen and crevice former utilized for the crevice corrosion test.

Figure 1 presents a composite image of the sample and the crevice former. To simulate the behavior of crevice corrosion at the location where the canister contacts the storage module support structure, we used specimens deposited with salt and wrapped with a crevice former to create a crevice configuration. The specimen’s thickness was precisely 3 mm. The chemical composition of the 304L SS utilized for this study is provided in Table 1. The sample was composed of 304L SS, and the crevice former was composed of polytetrafluoroethylene. Prior to the execution of the crevice corrosion examination, the surface of the specimen was subjected to a grinding process using 2000-grit abrasive paper.

The present study employed white emery as a means to simulate the accumulation of dust on the as-machined specimens during the testing of crevice corrosion induced by dust deposition on the canister surface. Figure 1 demonstrates the dimensions of the as-machined specimens, while the surface roughness was 4.9 μm. The as-machined samples were prepared from 304L SS, while the white emery was prepared from alumina. Each experimental condition was replicated.

**Figure 2:**
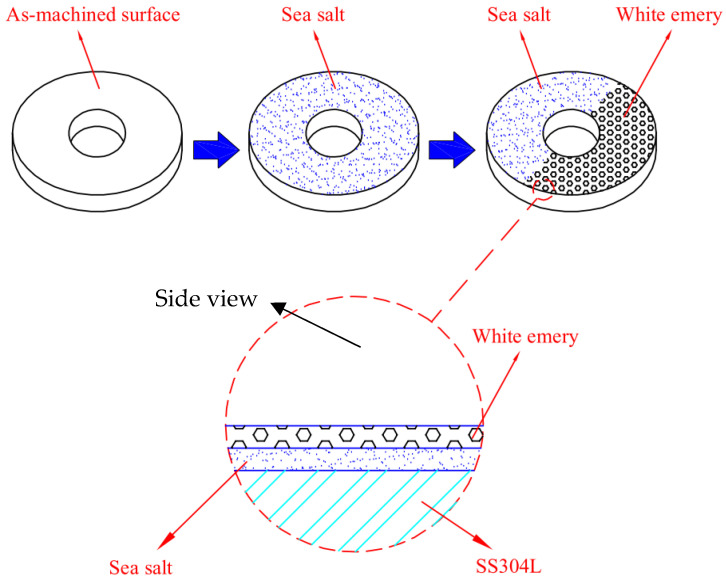
Schematic representation of the specimen sprayed with white emery and synthetic seawater [39].

Table 2 illustrates the chemical composition of the se salt utilized in the present research, which adheres to the standards outlined in ASTM D 1141-98 (2013) [38], as indicated under Formula a, Table X1.1. In the standard crevice corrosion test, the specimens were exposed to a salt spray using experimental equipment and synthetic seawater at a concentration of 3.5 wt%. Subsequently, the specimens were dried on a hot plate at 65 °C for a duration of 15 min. Each specimen’s mass difference was evaluated to determine the salt load in g/m^2^ after drying. The sample chloride deposition density was maintained at 0.1 g/m^2^ and 1 g/m^2^. The sample and the crevice former were affixed to each other with an M6 bolt, and torque was applied to 1.13 Nm to create the crevice configuration. Similarly, this research used white emery to imitate accumulated dust on the as-machined specimens’ surfaces to simulate crevice corrosion originating from dust deposition. A total of 0.5 g of white emery was uniformly dispersed via spray application around the circumference of the sample, rather than being fixed with a crevice former. Figure 2 [39] presents a schematic representation of the specimen after exposure to artificial seawater and white emery. All exposures were conducted within temperature- and humidity-controlled chambers, where the temperature was maintained at 35 °C or 45 °C and the relative humidity (RH) was set at 45%, 55%, or 70%. The duration of the exposures was 8000 h or 23,000 h, as detailed in Table 3. Metallographic procedures were utilized to prepare the specimens for microstructural analysis. The preparation of the samples included an ultrasonic cleansing step with deionized water, subsequent drying, and mounting in resin. Aluminum oxide powder was utilized in the polishing of the mounted specimens, which were then subjected to thorough examinations aimed at analyzing their microstructure and surface characteristics. In addition, a JSM-7100F field-emission SEM (JEOL, Tokyo, Japan) equipped with an EBSD detector (Oxford Instruments, Abingdon, UK) was utilized to investigate the SCC characteristics of the tested specimens. The kernel average misorientation (KAM) map and the strain countering map for the samples can be extracted by entering the EBSD map into the HKL-Channel 5 software (5.12.67.0, Oxford Instruments, Abingdon, UK) for data processing.

## 3. Results

### 3.1. Surface Morphology Examination

**Figure 3:**
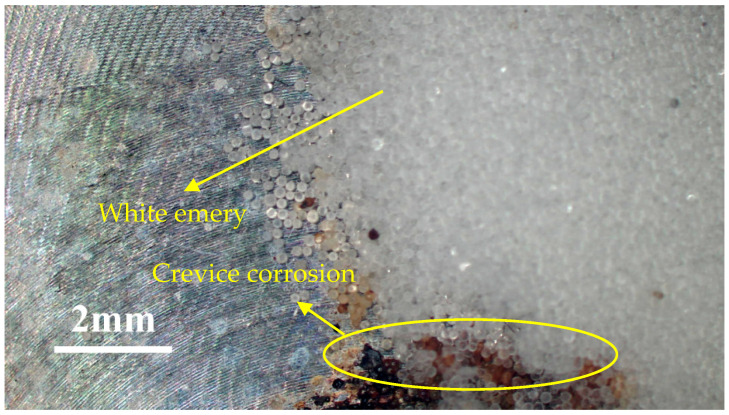
Macrographs of specimens subjected to white emery spray and deposited at a 1 g/m^2^ chloride concentration at a temperature of 45 °C and a relative humidity of 70%.

**Figure 4:**
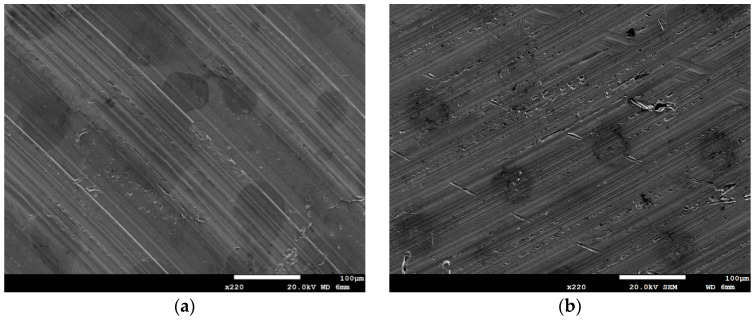
SEM micrographs of specimens subjected to white emery spray and deposited at a 0.1 g/m^2^ chloride concentration and 70% RH after 8000 h of testing at: (**a**) 35 °C, and (**b**) 45 °C.

**Figure 5:**
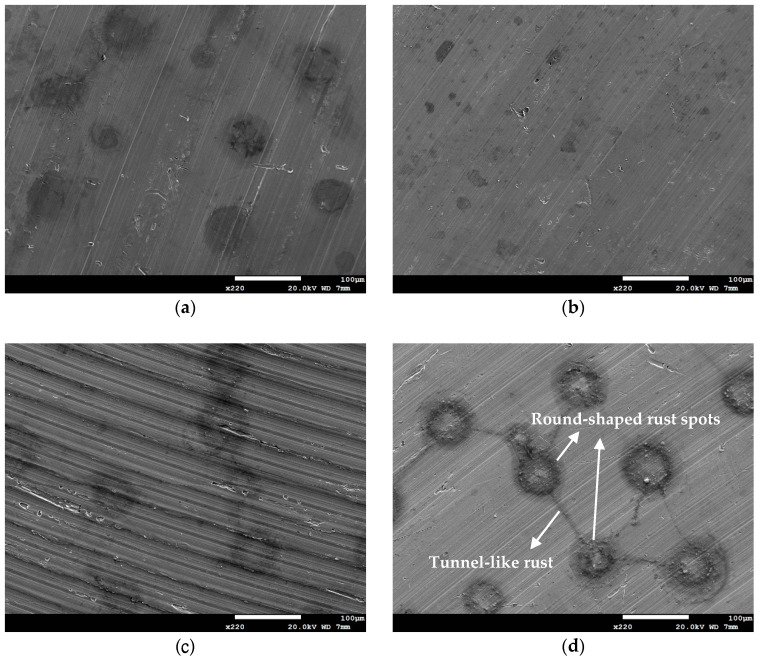
SEM micrographs of specimens subjected to white emery spray and deposited at a 0.1 g/m^2^ chloride concentration after 23,000 h of testing at: (**a**) 35 °C and 70%RH, (**b**) 45 °C and 45% RH, (**c**) 45 °C and 55% RH, and (**d**) 45 °C and 70% RH.

As demonstrated in Figure 3, the 304L specimen that was sprayed with 0.5 g of white emery and exposed to 1 g/m^2^ chloride at a temperature of 45 °C and a relative humidity of 70% reveals the presence of a corrosion band along the semicircular deposit. The corrosion products form a belt approximately 1 mm in width at the deposit edge, indicating localized crevice corrosion beneath the white emery layer. Figure 4 and Figure 5 illustrate the SEM images of the specimens that were subjected to the white emery spray and a 0.1 g/m^2^ chloride concentration. The SEM micrographs demonstrate that, under conditions of a lower salt load (0.1 g/m^2^), there is a marked increase in the corrosion severity from 35 °C to 45 °C at 70% RH. Furthermore, the initial appearance of rust spots is observed at a relative humidity of at least 55%, and their progression to interconnected “tunnels” is evident only at 70% RH (see Figure 5d). Notably, at a temperature of 35 °C, even after 23,000 h of exposure, only shallow pits are observed, suggesting a different susceptibility to rust under these conditions.

**Figure 6:**
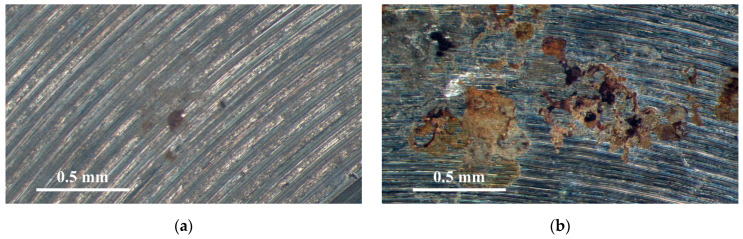
Macrographs of specimens subjected to white emery spray and deposited at a 1 g/m^2^ chloride deposition density and 70% RH after 8000 h of testing at: (**a**) 35 °C, and (**b**) 45 °C.

**Figure 7:**
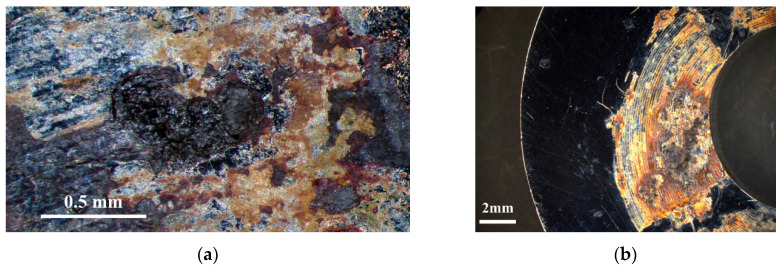
Macrographs of specimens enveloped within the crevice former and deposited at a 1 g/m^2^ chloride concentration and 70% relative humidity after 8000 h of testing at: (**a**) 35 °C, and (**b**) 45 °C.

Figure 6 and Figure 7 are the macrographs of the 304L stainless steel specimens sprayed with white emery and wrapped with a crevice former at two different temperature levels; all specimens were deposited at a chloride concentration of 1 g/m^2^. Figure 6a and Figure 7a demonstrate the presence of crevice corrosion on the specimens subjected to white emery spray and enveloped within the crevice former under conditions of 35 °C. The specimens wrapped with a crevice former, as shown in Figure 7a, exhibit larger corrosion pits than those sprayed with white emery, shown in Figure 6a. Significant corrosion of the samples is revealed in Figure 7b, with products of corrosion on exposed surfaces. The specimen’s appearance indicates a decrease in brightness, with a surface color that appears yellowish-brown. Furthermore, the areas of rust on the specimens subjected to white emery spray and wrapped with a crevice former increased as the temperature increased, as shown in Figure 6 and Figure 7. It has been demonstrated that exposure to constant humidity, in conjunction with rising temperatures, results in accelerated diffusion and electrochemical kinetics, which, in turn, gives rise to an enhancement in corrosion rates [33,40]. Moreover, it appears that the specimens enveloped within the crevice former, Figure 7a,b, are more corroded than those sprayed with white emery, Figure 6a,b, at both temperature levels.

### 3.2. Investigation of EDS and EBSD

**Figure 8:**
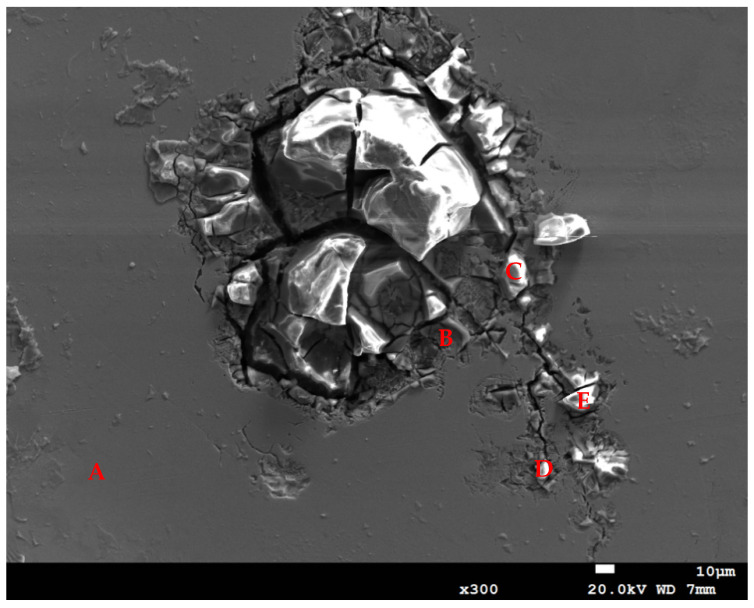
EDS examination of corrosion pits on the specimen enveloped within the crevice former and deposited at a 1 g/m^2^ chloride concentration at 35 °C and 70% RH (EDS analysis results of points A–E are listed in Table 4).

Figure 8 demonstrates the EDS examinations of the corrosion pits in adjacent cracks on the specimen wrapped with a crevice former and deposited with a 1 g/m^2^ chloride deposition density at 35 °C and 70% RH. The findings of the EDS analysis of the corrosion pits are presented in Table 4. At point B, the iron content was significantly higher and the oxygen content was lower in comparison to points C and E. This observation suggests that the oxide product was peeled off, thereby unveiling the metal surface. Furthermore, the chlorine and sulfur content of point A, the matrix of stainless steel, was not detectable due to the extremely low concentration levels. Trace quantities of Cl were observed at points B, C, D, and E, which were located in the corrosion products. It was determined that points B, C, D, and E contained higher concentrations of sulfur in comparison to point A, probably due to the sulfates included in the synthesized seawater.

Crevice corrosion initiation is significantly affected through the presence of chloride ions. The critical crevice solution (CCS) mechanism suggests that the onset of crevice corrosion is dependent on the accumulation of corrosive ions, particularly chloride (Cl^−^), at the crevice sites. This phenomenon has been observed to result in the onset of localized corrosion, characterized by its notably aggressive nature. The severity of this localized corrosion has the capacity to exert a substantial detrimental effect on stainless steel’s passive film [41]. Compared to points C and E, point B contains higher concentrations of iron but lower concentrations of oxygen. This suggests that the oxide product was stripped off, exposing the surface of the metal.

**Figure 9:**
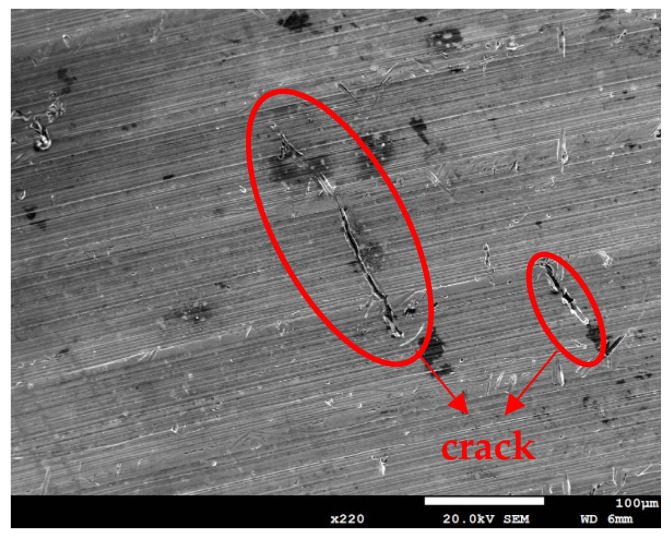
SEM morphologies of SCC on specimens subjected to white emery spray and deposited at a 0.1 g/m^2^ chloride concentration after 8000 h of testing at a temperature of 45 °C and a relative humidity of 70%.

**Figure 10:**
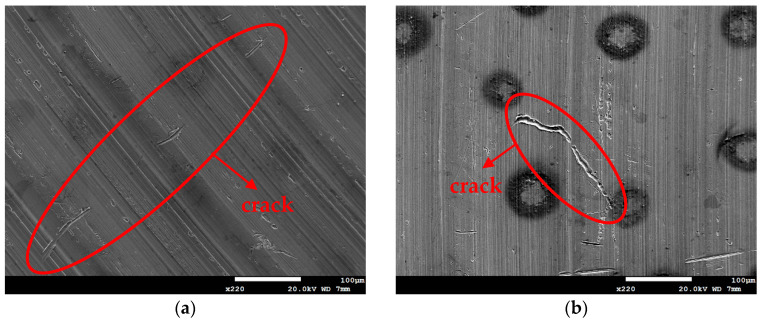
SEM morphologies of SCC on specimens subjected to white emery spray and deposited at a 0.1 g/m^2^ chloride deposition density and 70% RH after 23,000 h of testing at: (**a**) 35 °C, and (**b**) 45 °C.

Figure 9 and Figure 10, respectively, show SEM micrographs that reveal the morphology of SCC on the specimens sprayed with white emery and deposited at a chloride deposition density of 0.1 g/m^2^ and 70% RH at 45 °C for 8000 h and at 35 °C and 45 °C for 23,000 h. These findings indicate that SCC cracks initiate only under sufficiently aggressive conditions (70% RH). Following an exposure duration of 8000 h at a temperature of 45 °C, the formation of discontinuous microcracks becomes evident in the region adjacent to the rust spots. These microcracks exhibit a greater length when compared to those observed in specimens that were exposed for 23,000 h at a temperature of 35 °C. The extension of the 0.1 g/m^2^ tests to 23,000 h at 45 °C results in the transformation of these microcracks into an almost continuous crack. This finding suggests that temperature exerts a more significant influence on crack morphology than exposure duration under low salt loading conditions.

**Figure 11:**
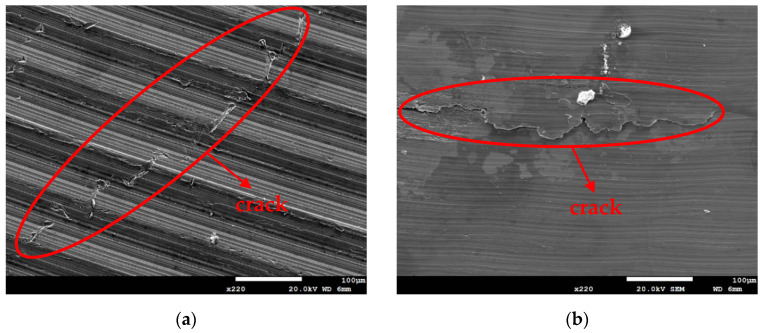
SEM morphologies of SCC on specimens subjected to white emery spray and deposited at a 1 g/m^2^ chloride deposition density and 70% RH after 8000 h of testing at: (**a**) 35 °C, and (**b**) 45 °C.

**Figure 12:**
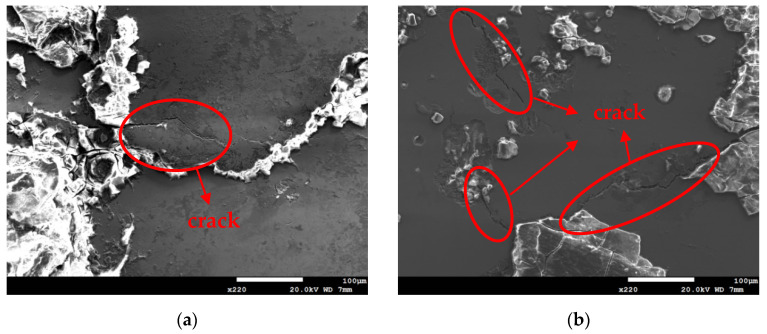
SEM morphologies of SCC on specimens enveloped within the crevice former and deposited at a 1 g/m^2^ chloride deposition density and 70% RH after 8000 h of testing at: (**a**) 35 °C, and (**b**) 45 °C.

Figure 11 and Figure 12 are SEM micrographs showing the morphology of stress corrosion cracking on specimens subjected to white emery spray and wrapped with a crevice former at two different temperature levels. All of the specimens were deposited with a 1 g/m^2^ chloride deposition density. Figure 11a shows discontinuous SCC cracks on the specimen tested at 35 °C, while Figure 11b exhibits distinct cracks of SCC on specimens exposed to 45 °C. Similarly, the sample exposed to 45 °C, as illustrated in Figure 9, exhibited discrete SCC cracks on the specimens deposited with a 0.1 g/m^2^ chlorine deposition density, while Figure 11b demonstrates continuous cracks of SCC on the specimens deposited with a 1 g/m^2^ chloride deposition density. Figure 9, Figure 10a, and Figure 11a illustrate short, shallow cracks on the specimens, in contrast to Figure 11b and Figure 12a,b, which illustrate long, deep cracks on the specimens. According to the assumption, microcracks first nucleate non-sequentially at appropriate locations and under appropriate conditions, and the process of crack growth is characterized by the formation of microcracks, which subsequently coalesce through the rupture of ligaments that connect them, resulting in the formation of a primary crack [35,36].

**Figure 13:**
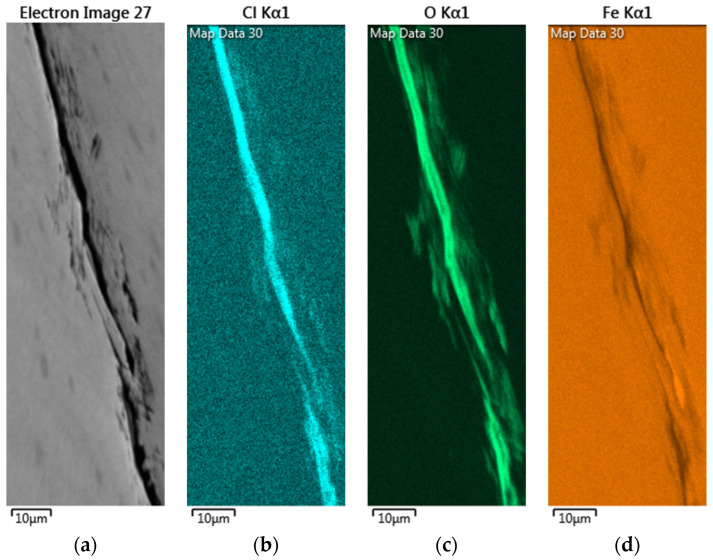
EDS mapping for the crack areas of a specimen enveloped within the crevice former and deposited with a chloride deposition density of 1 g/m^2^ at 35 °C and 70% RH: (**a**) IQ (Image quality) figure, (**b**) Cl mapping, (**c**) O mapping, and (**d**) Fe mapping.

Based on the aforementioned assumption, the specimens subjected to white emery spray and deposited with a 0.1 g/m^2^ chloride deposition density and exposed to 45 °C, and deposited with a 1 g/m^2^ chloride deposition density and exposed to 35 °C were subjected to 8000 h of testing. The specimens exhibited the presence of a few shallow and short cracks, which could be attributed to the limited number of chlorine ions transported to the sites of crevices in the specimens. Owing to the extremely low chloride concentration level of the specimens tested, it is imperative to establish an elevated-temperature environment to enhance the transportation of a sufficient quantity of Cl^−^ to the site of crevice formation, thereby facilitating cracking nucleation. The maps of chlorine acquired by EDS mapping, as demonstrated in Figure 13b, provide the supporting evidence for the above argument. This will be discussed in detail below. After testing for 8000 h, no cracks of SCC were investigated on the specimens subjected to white emery spray and tested at a temperature of 35 °C, whereas cracks were observed on the specimens tested at 45 °C, a higher-temperature environment, and on the specimens deposited with a chloride deposition density of 0.1 g/m^2^, as shown in Figure 4 and Figure 9. The occurrence of SCC cracks was observed at a temperature of 35 °C only when the test duration was increased to 23,000 h, as illustrated in Figure 10. The chloride deposition density of 0.1 g/m^2^ was likely insufficient to initiate stress corrosion cracking on the specimens subjected to the decreased temperature and test duration. The increased volume of corrosion products likely generated a localized stress state beneath the white emery, thereby functioning similarly to a crevice former. This phenomenon is believed to be a contributing factor to the development of SCC. Moreover, an investigation of the crack length revealed a tendency for it to increase with temperature when comparing Figure 12a,b.

Figure 13 presents the EDS mapping for the crack regions of the specimens wrapped with a crevice former and deposited with a chloride deposition density of 1 g/m^2^ at 35 °C and 70% RH. The crack region is rich in chlorine and oxygen, as illustrated in Figure 13b,c, while it is depleted of iron, as demonstrated in Figure 13d. The presence of corrosion products of iron in the vicinity of the crack region suggests that the process of corrosion resulted in a depletion of iron at the site of the crack. According to the critical crevice solution (CCS) model, the depletion of oxygen within the crevice can lead to the acidification of the solution at crevice sites. Consequently, breakdown of the passive film and initiation of crevice corrosion can occur. This model emphasizes the accumulation of aggressive ions (e.g., Cl^−^) at crevice sites, followed by depassivation and subsequent base metal dissolution [17]. The enhanced chloride concentration has been demonstrated to lower local pH and acidify the solution. Consequently, the passive film is destabilized and localized crevice corrosion is initiated, thereby promoting crack formation.

**Figure 14:**
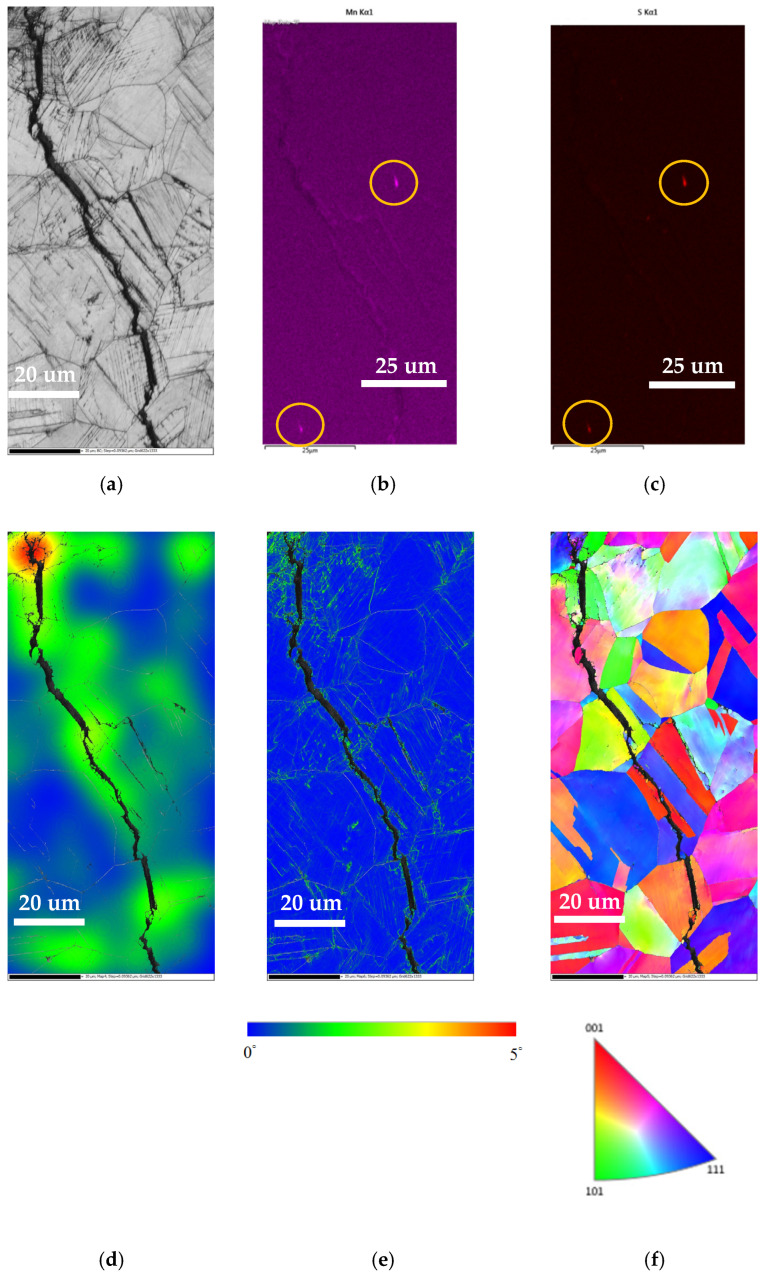
EBSD maps and EDS mapping for the crack areas of the specimen enveloped within the crevice former and deposited at a 1 g/m^2^ chloride concentration exposed to 35 °C and 70% RH: (**a**) IQ figure, (**b**) Mn mapping, (**c**) S mapping, (**d**) strain countering map, (**e**) KAM map, and (**f**) inverse pole figure (IPF) map.

Figure 14 illustrates the EBSD and EDS maps for the crack areas of the specimen enveloped within the crevice former and deposited with a 1 g/m^2^ chloride deposition density exposed to 35 °C and 70% relative humidity. Figure 14a,f demonstrate that the specimens were cracked by a TGSCC mode, which is perfectly consistent with previously reported observations of stainless steel induced by chlorides [22]. Figure 14b and Figure 14c, respectively, indicate enriched manganese and sulfur at the top right and bottom left of this figure. The distribution of manganese and sulfur within the metal is related to the existence of non-metallic MnS inclusions. The strain countering map and kernel average misorientation (KAM) map (Figure 14d,e) illustrate that the stress is predominantly concentrated in the vicinity of the crack areas. In addition, as illustrated in Figure 14d, high plastic strain is observed in the regions adjacent to the cracks, suggesting a probable correlation with the plastic strain present at the tip of the cracks. The propagation of cracks was found to be associated with the degree of plastic strain present at the tips of the cracks. EBSD analyses confirm that SCC proceeds via a TGSCC mechanism, consistent with chloride-induced cracking in 304L SS [22]. The presence of MnS inclusions, as identified through the use of EDS mapping of Mn and S, has been demonstrated to serve as a stress concentrator. These inclusions have been observed to initiate micro-pits, which subsequently evolve into visible cracks. Strain contouring analysis reveals that plastic deformation localizes at the crack front (see Figure 14d), thereby accelerating film rupture and chloride ingress. Additionally, kernel average misorientation (KAM) mapping reveals elevated subgrain rotation densities in the immediate vicinity of the crack (see Figure 14e), suggesting the accumulation of geometrically necessary dislocations (GNDs). These GNDs not only accommodate strain but also promote crack tip branching under cyclic film breakdown.

## 4. Discussions

Following an 8000 h test, SCC cracks were observed exclusively on the specimen subjected to white emery spray and tested at a temperature of 45 °C and a 0.1 g/m^2^ chloride concentration. Increasing the chloride concentration to 1 g/m^2^ resulted in a higher level of corrosion and a greater severity of SCC for the specimens enveloped within the crevice former (see Figure 7 and Figure 12). This was observed in comparison to the specimens subjected to white emery spray (see Figure 6 and Figure 11) at both temperature levels. Factors influencing crevice corrosion initiation include the existence of aggressive chlorine ions, the ambient temperature, and the crevice geometry. Generally, the severity of environmental conditions is directly proportional to the tightness of the crevice and therefore to the probability of corrosion initiation [42]. The presence of deposits, such as dust, on the metal surface has been shown to act as a crevice former. The testing of the PTFE crevice former was conducted by maintaining the specimen and the crevice former in a fixed position through the application of a torque of 1.13 Nm. Conversely, the testing of the white emery deposit was conducted with no external force applied to the sample and the white emery for fixation. Consequently, the crevice was tighter when the specimen was enveloped within the crevice former than when it was sprayed with white emery. Nevertheless, an increase in surface roughness indicates the presence of deeper grooves on the material surface, where a higher concentration of chloride ions occurs, resulting in early corrosion initiation. The roughness of the test specimen was measured at 0.5 μm in the testing of the PTFE crevice former, while the as-machined specimen exhibited a surface roughness of 4.9 μm in the testing of the white emery deposit.

In summary, the 8000 h test demonstrated that an enhancement in the effect of surface roughness was observed when the chloride deposition density for the samples was 0.1 g/m^2^. Consequently, the presence of SCC cracks was exclusively observed in specimens that were subjected to white emery spray and tested at a temperature of 45 °C. However, an increase in the chloride deposition density to 1 g/m^2^ resulted in crevice tightness becoming the predominant factor, surpassing surface roughness. This observation indicates that specimens enveloped within the crevice former revealed higher levels of corrosion and severity of surface cracks compared to those subjected to white emery spray at both temperature levels. The hypothesis suggested that crevice corrosion is affected by two interrelated factors: surface roughness and crevice tightness. It can be concluded that the SCC initiation of 304L stainless steel was the result of the combined effects of surface roughness and crevice tightness.

## 5. Conclusions

This study analyzes the characteristics of crevice corrosion behavior of 304L SS subjected to combined ambient temperatures of 35 °C and 45 °C, relative humidities of 45%, 55%, and 70%, chloride concentrations of 0.1 g/m^2^ and 1 g/m^2^, and test durations of 8000 h and 23,000 h. The specimens were tested with PTFE crevice formers wrapped on the grounded surfaces and white emery deposited on the surface of the as-machined material. The conclusions of the present study are summarized below:

(1) In the 8000 h tests, discontinuous SCC cracks were observed only in the specimens subjected to white emery spray at 45 °C and 70% relative humidity with a 0.1 g/m^2^ chloride deposition density; at a 1 g/m^2^ chloride deposition density, the PTFE crevice former test specimens developed continuous SCC cracks under identical conditions. The extension of the test to 23,000 h at a 0.1 g/m^2^ chloride deposition density resulted in the formation of continuous cracks, even under the presence of white emery deposits. This observation indicates that the SCC initiation threshold is located within the range of 8000 h to 23,000 h.
(2) When environmental conditions are constant, the duration of the tests is observed to affect the morphology of SCC cracks on the specimens. The cracks transition from a discontinuous to a continuous mode as the duration of the test increases. This finding suggests that the morphologically observed evolution from discrete to interconnected cracks is driven by a synergistic effect of increasing local stress intensity under crevice confinement, progressive chloride ion enrichment leading to passive film breakdown, and microstructural degradation marked by dislocation accumulation.
(3) The specimens with a chloride deposition density of 1 g/m^2^ experienced significant crevice corrosion exclusively in regions where white emery was deposited.
(4) The 304L stainless steel specimens exhibited cracking under TGSCC mode, which was confirmed by the EBSD analysis results.

## Figures and Tables

**Table 1:** Chemical composition of the 304L stainless steel utilized in this study.

Element	C	Si	S	Cr	Ni	Mn	Fe
Wt%	0.017	0.450	0.029	18.000	9.000	1.540	Bal.

**Table 2:** Chemical composition of the sea salt used in this work.

Composition	MgCl_2_	NaCl	Na_2_SO_4_	KCl	CaCl_2_	KBr	NaHCO_3_	SrCl_2_	NaF	H_3_BO_3_
wt%	26.460	58.490	9.750	1.645	2.765	0.238	0.477	0.095	0.007	0.071

**Table 3:** Test conditions of the specimens.

Parameter	Unit	Value
Temperature	°C	35, 45
Relative humidity	%	45, 55, 70
Chloride deposition density	g/m^2^	0.1, 1
Test duration	Hours	8000, 23,000
Testing method for sample surface	N/A	White emery deposition, crevice former

**Table 4:** EDS results on the selected location of Figure 8 in weight percentage (wt%).

Location	Fe	Cr	Ni	Mn	O	S	Cl
A	69.9	18.9	8.00	1.80	1.40	0.00	0.00
B	23.6	46.5	3.60	1.50	23.0	1.50	0.30
C	14.1	35.6	3.90	0.50	43.2	2.30	0.50
D	19.6	28.5	2.40	0.70	46.2	2.30	0.40
E	15.1	29.6	3.90	0.60	47.8	2.80	0.30

## Data Availability

The original contributions presented in this study are included in the article. Further inquiries can be directed to the corresponding author.
